# Dual Roles of Astrocyte-Derived Factors in Regulation of Blood-Brain Barrier Function after Brain Damage

**DOI:** 10.3390/ijms20030571

**Published:** 2019-01-29

**Authors:** Shotaro Michinaga, Yutaka Koyama

**Affiliations:** 1 Laboratory of Pharmacology, Faculty of Pharmacy, Osaka Ohtani University, 3-11-1 Nishikiori-Kita, Tondabayashi, Osaka 584-8540, Japan; mitinasy@osaka-ohtani.ac.jp; 2 Laboratory of Pharmacology, Kobe Pharmaceutical University, 4-19-1 Motoyama-Kita Higashinada, Kobe 668-8558, Japan

**Keywords:** astrocytes, blood-brain barrier, endothelial cells, tight junction

## Abstract

The blood-brain barrier (BBB) is a major functional barrier in the central nervous system (CNS), and inhibits the extravasation of intravascular contents and transports various essential nutrients between the blood and the brain. After brain damage by traumatic brain injury, cerebral ischemia and several other CNS disorders, the functions of the BBB are disrupted, resulting in severe secondary damage including brain edema and inflammatory injury. Therefore, BBB protection and recovery are considered novel therapeutic strategies for reducing brain damage. Emerging evidence suggests key roles of astrocyte-derived factors in BBB disruption and recovery after brain damage. The astrocyte-derived vascular permeability factors include vascular endothelial growth factors, matrix metalloproteinases, nitric oxide, glutamate and endothelin-1, which enhance BBB permeability leading to BBB disruption. By contrast, the astrocyte-derived protective factors include angiopoietin-1, sonic hedgehog, glial-derived neurotrophic factor, retinoic acid and insulin-like growth factor-1 and apolipoprotein E which attenuate BBB permeability resulting in recovery of BBB function. In this review, the roles of these astrocyte-derived factors in BBB function are summarized, and their significance as therapeutic targets for BBB protection and recovery after brain damage are discussed.

## 1. Introduction

**Figure 1:**
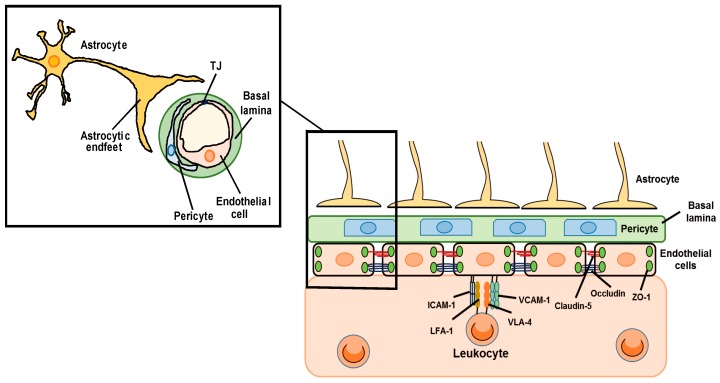
The BBB comprises endothelial cells, pericytes and astrocytes. The low permeability to serum components results from dense formation of TJs between brain microvascular endothelial cells. TJs comprise TJ-related proteins including claudin-5, occludin and ZO-1. Astrocytes produce several factors that modulate the expression of the TJ-related proteins and regulate paracellular transport across vascular endothelial cells. In addition, astrocyte-derived factors affect the expression of endothelial ICAM-1 and VCAM-1, which interact with VLA-4 and LFA-1 in leukocytes. Increased ICAM-1 and VCAM-1 expression promotes leukocyte infiltration into the CNS.

The blood–brain barrier (BBB) is a biological and functional barrier in the central nervous system (CNS), and comprises various types of cells including endothelial cells, pericytes and astrocytes (Figure 1). The BBB limits the influx of intravascular contents including serum proteins, blood cells and toxic substances into the cerebral parenchyma, and pumps out cerebral waste materials [1]. The BBB also expresses a range of transporters essential for movement of amino acids and glucose into the cerebral parenchyma to support the function and survival of brain cells. These static barrier functions and transportation systems of the BBB are regulated by endothelial cells, pericytes and astrocytes. Under physiological conditions, BBB permeability is strictly regulated by cell–cell interactions and cell-derived bioactive factors [2]. The static barrier function depends on endothelial tight junctions (TJs) and the basal lamina (Figure 1). The TJ is formed by TJ-related proteins including claudin (CLN), occludin (OCLN) and zonula occluden (ZO) [3]. The basal lamina is a layer of extracellular matrix known as the basement membrane, which consists of collagen, laminin and fibronectin. Astrocytes exist around cerebral microvessels and control BBB functions via astrocyte-derived factors and astrocytic terminal processes termed endfeet. Astrocytic endfeet express the potassium channel, Kir4.1, and aquaporin-4, which support the BBB function by controlling the ion and water balance [4]. BBB is also responsible for the regulation of leukocyte infiltration into the CNS (Figure 1). During the process of leukocyte infiltration, cell adhesion molecules (CAMs) on endothelial cells, including vascular cell adhesion molecule-1 (VCAM-1) and intercellular adhesion molecule-1 (ICAM-1), have roles in leukocyte adhesion. Endothelial ICAM-1 and VCAM-1 interact with very late antigen-4 (VLA-4) and lymphocyte function-associated antigen 1 (LFA-1) in leukocytes, causing firm adhesion of endothelial cells and leukocytes. The expression of ICAM-1 and VCAM-1 in brain vascular endothelial cells is regulated by chemokines and inflammatory cytokines produced by astrocytes [5,6]. In this way, astrocytes can affect leukocyte infiltration into the CNS.

**Figure 2:**
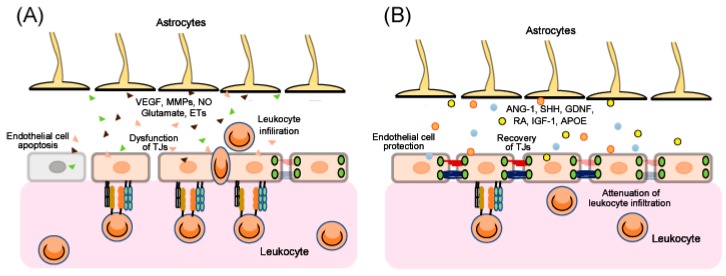
Dual roles of astrocyte-derived factors in the regulation of BBB functions. In brain disorders, astrocytes release various kinds of extracellular signaling molecules. (**A**) Vascular permeability factors: Astrocyte-derived vascular endothelial growth factors (VEGFs), matrix metalloproteinases (MMPs), nitric oxide (NO), glutamate and endothelins (ETs) cause endothelial apoptosis and downregulation of TJ-related proteins, resulting in BBB disruption. Some of these factors also upregulate endothelial CAMs, which induce leukocyte transmigration. (**B**) Vascular protective factors: Astrocyte-derived angiopoietin-1 (ANG-1), sonic hedgehog (SHH), glial-derived neurotrophic factor (GDNF), retinoic acid (RA), insulin-like growth factor-1 (IGF-1) and apolipoprotein E (APOE) protect endothelial cells from apoptosis and promote recovery of TJ function. Some of these factors also decrease endothelial CAMs’ expression and reduce leukocyte infiltration.

After traumatic brain injury (TBI), ischemia and various other CNS disorders, the functions of the BBB can be disrupted [7,8,9,10,11], and the resulting excessive BBB permeability causes secondary damage including brain edema and inflammatory injury. Therefore, BBB protection and recovery are essential for reducing the progression of brain damage. Apoptosis of endothelial cells and/or dysfunction of endothelial TJs results in disruption of BBB function (Figure 2). Upregulation of CAMs on endothelial cells accelerates leukocytes crossing the BBB (Figure 2). Further, after injury, astrocytes are converted from a resting form to a reactive form, and several astrocyte-derived factors induce endothelial cell apoptosis and decrease expression of endothelial TJ-related proteins, leading to aggravation of BBB disruption (Figure 2). By contrast, some astrocyte-derived factors can protect endothelial cells and enhance TJ reassembly, leading to BBB recovery (Figure 2). In addition, several astrocyte-derived factors also regulate CAMs on endothelial cells and control leukocyte crossing the BBB (Figure 2).

Therefore, the appropriate control of astrocyte-derived factors to reduce BBB damage and promote BBB recovery is becoming of increasing interest as a therapeutic strategy after brain damage. In this review, we describe several key astrocyte-derived factors involved in BBB function, and discuss the significance of these factors as novel therapeutic targets for BBB recovery after brain damage.

## 2. The Pathogenesis of BBB Disruption

BBB disruption causes extravasation of intravascular fluid and excessive infiltration of leukocytes including neutrophils, monocytes and lymphocytes into the cerebral parenchyma, resulting in brain edema and inflammatory injury, respectively. BBB disruption has been confirmed in patients with TBI and ischemic stroke [7,8], and is associated with the progression of various CNS disorders including Alzheimer’s disease, multiple sclerosis and Parkinson’s disease [9,10,11]. BBB disruption has also been reproduced in various models of brain disorders [12,13,14,15].

The mechanisms underlying BBB disruption include direct injury to vascular endothelial cells in the core area and excessive BBB permeability in the peri-core area (Figure 2). The direct injury induces an irreversible BBB disruption due to the death of BBB cells. For example, endothelial cell apoptosis has been reported in ischemic animal models and following oxygen-glucose deprivation in vitro, resulting in a pathological increase in BBB permeability [16]. Brain endothelial cell apoptosis has also been reported in TBI model animals, including activation of the c-Jun N-terminal kinase, p38 mitogen-activated protein kinase and caspase-3 pathways [17].

In the peri-core area of brain injury, excessive BBB permeability can also result from increases in paracellular transport caused by dysfunction of endothelial TJs (Figure 2). For example, decreases in CLN-5, OCLN and ZO-1 were observed in ischemic stroke and TBI animal models [18,19,20]. Argaw et al. [21] and Wang et al. [22] have reported decreases in TJ-related proteins in animal models of CNS inflammation such as multiple sclerosis. Furthermore, phosphorylation of TJ-related proteins caused their detachment, leading to TJ dysfunction [3,23]. These observations suggest that protection of endothelial cells and promotion of recovery of endothelial TJ-related protein function are therapeutic targets for BBB disruption, which may reduce the pathogenesis of various CNS disorders and brain injuries.

The leukocytes that cross the BBB also accumulate in the damaged brain. The expression of VCAM-1 and ICAM-1 on endothelial cells was increased in experimental animals after brain damage [24,25,26], and the increased endothelial CAMs potentiated binding to adhesion molecules in leukocytes, such as VLA-4 and LFA-1. The interaction of these adhesion molecules is a key process for leukocytes crossing the BBB. The infiltration of neutrophils, monocytes and lymphocytes was observed around the injured core upon experimental brain damage [26,27,28,29]. Accumulation of leukocytes has also been shown in patients with TBI [30]. Moreover, astrocyte-derived chemokines, including monocyte chemotactic protein-1, and macrophage inflammatory proteins accelerate infiltration of leukocytes [31,32].

## 3. Regulation of BBB Function by Astrocyte-Derived Factors

Several studies suggest dual roles for astrocytes in the control of BBB function. Eilam et al. [33] revealed that loss of astroglial connections with blood vessels caused BBB disruption in an animal model of multiple sclerosis. By contrast, Begum et al. [13] showed that selective knock-out of the astrocytic Na^+^/H^+^ exchanger isoform 1 reduced astrogliosis after ischemic stroke in mice, with a resulting decrease in cerebral vessel damage and improved BBB function. Chiu et al. [14] also reported that ethyl-1-(4-(2,3,3-trichloroacrylamide)phenyl)-5-(trifluoromethyl)-1H-pyrazole-4-carboxylate decreased the pathological activation of astrocytes and reduced BBB destruction in intracerebral hemorrhage model rats. Overall, these studies imply that appropriate regulation of astrocyte function is required to attenuate BBB disruption and promote BBB function after brain injury. Astrocyte-derived factors are known to be responsible for both BBB disruption and repair (Figure 2). Below, we describe a range of astrocyte-derived factors and their roles in BBB disruption.

### 3.1. The Vascular Permeability Factors

#### 3.1.1. Vascular Endothelial Growth Factor

Vascular endothelial growth factors (VEGFs) including VEGF-A, -B, -C, -D, -E and-F are known as an angiogenetic factor and exert angiogenetic functions via VEGF receptor-1 (VEGFR-1) and -2 (VEGFR-2), which are tyrosine kinase receptors expressed in endothelial cells. The activation of endothelial VEGF/VEGFR signal leads to endothelial proliferation and differentiation for angiogenesis [34]. On the other hands, VEGFs are also well-established to promote BBB permeability. For example, exogenous treatment with VEGF in animals and in cultured brain microvessel endothelial cells caused increased BBB permeability [35,36], while treatment with an anti-VEGF neutralizing antibody reduced BBB leakage (Evans blue staining) in cerebral ischemia/reperfusion [37] and focal TBI by fluid percussion injury (FPI) [12] animal models. Inhibition of VEGF signaling by SU5416, a VEGF receptor-2 inhibitor, and specific VEGF receptor-2 knockdown, also reduced BBB disruption after permanent ischemic damage by thrombosis [38]. Furthermore, VEGF was reported to protect against endothelial cell apoptosis under hypoglycemic conditions [39]. On the other hand, VEGF downregulated the expression of TJ-related proteins on brain endothelial cells [35,40]. Therefore, VEGF enhances BBB permeability by decreasing TJ-related proteins. In animal models of multiple sclerosis, normal expressions for VCAM-1 and ICAM-1 were displayed in the inactivation of astrocyte-specific VEGF-A mice, and the inactivation of astrocyte-specific VEGF-A reduced lymphocyte infiltration [40]. In human umbilical vascular endothelial cells (HUVECs), VEGF also induced ICAM-1 and VCAM-1 expressions, and induced leukocyte adhesion to HUVECs [41].

Following brain injury and in various CNS disorders, induction of VEGF was observed in reactive astrocytes although it is also produced in various types of cells in CNS. Several studies indicate the involvements of astrocytic VEGF for BBB disruption. Argaw et al. [40] reported that astrocytes expressed VEGF-A, while inactivation of a144strocyte-specific VEGF-A reduced BBB disruption in animal models of multiple sclerosis. Chapouly et al. [15] also reported VEGF-A expression on reactive astrocytes in human multiple sclerosis and experimental animal models, while blockade of VEGF-A by cavtratin, a selective inhibitor of VEGF-A signaling, protected against BBB disruption. Finally, we previously reported an increase in VEGF-A expression in astrocytes after brain damages in mice, and that blockade of VEGF-A using antibodies alleviated the BBB disruption [12]. In patients with brain damages including TBI and ischemic stroke, the increase of VEGF level was observed and suggested the relationships with degree of severity [42,43,44].

#### 3.1.2. Matrix Metalloproteinases

MMPs are zinc-endopeptidases which degrade endothelial TJ-related proteins and extracellular matrix (ECM) molecules including collagen, laminin and fibronectin. The degradation of ECM and TJ-related proteins are essential processes for angiogenesis while accelerating BBB permeability. In patients with TBI, elevation of MMPs in cerebrospinal fluid and blood was indicated [43,45,46]. Chen et al. [47] found that overexpression of MMP-9 caused degradation of CLN-5 and OCLN, resulting in endothelial barrier disruption, while in experimental animals of cerebral ischemia/perfusion, the MMP-induced reduction of TJ-related proteins resulted in BBB disruption [48,49]. Guo et al. [50] also reported that MMP-9 activity was responsible for endothelial cell apoptosis following subarachnoid hemorrhage in rats. Moreover, the excessive activation of MMP-2 and MMP-9 led to cellular damage in cerebral endothelium after hypoxia-reoxygenation [51]. The beneficial effects of MMP inhibition on BBB disruption were also examined in experimental animal models. For example, blocking MMP activation or MMP-9 knock-out (KO) prevented degradation of CLN-5 and OCLN, and attenuated BBB disruption, in cerebral ischemia/reperfusion animal models [52,53]. In focal TBI animals by FPI, MMP-9 inhibition also reduced BBB disruption [12]. Moreover, blockade of MMP-9 activity by Ro32–3555, a broad spectrum MMP inhibitor reduced transmigration of neutrophils and monocytes in an in vitro model of CNS tuberculosis [54]. MMP inhibitors also regulated inflammatory cell migration by reducing ICAM-1 and VCAM-1 expression in lung tissues in asthma model animals [55]. Therefore, regulation of ICAM-1 and VCAM-1 expressions by MMP may be also involved in infiltration of leukocytes in CNS.

MMPs are produced in various types of cells in CNS. In experimental animal models of brain injury, the expression of MMPs was also observed in astrocytes. Jiang et al. [56] found that reactive astrocytes released MMP-2 and MMP-9, while in amyloid precursor protein/presenilin 1 transgenic mice, MMP-2 and MMP-9 immunoreactivities were selectively increased in activated astrocytes [57]. Astrocytic MMP-9 activation also compromised the BBB and exacerbated intracerebral hemorrhage in animal models [58]. Finally, we confirmed the induction of MMP-9 in astrocytes in TBI mice by FPI, and found that inhibition of MMP-9 attenuated the TBI-induced BBB disruption [12].

#### 3.1.3. Nitric Oxide

Nitric oxide (NO) is a potent vasodilator and plays a role in neurovascular coupling by regulation of blood flow for neuronal activity [59]. NO is synthetized from L-arginine by NO synthase (NOS). There are three NOS isoforms, including neuronal NOS (NOS-1), inducible NOS (NOS-2) and endothelial NOS (NOS-3). NOS-1 and NOS-3 are constitutive and regulate endothelial cell functions under normal conditions, while NOS-2 is increased following injury to promote the inflammatory reaction. Various studies have also shown that astrocytes can produce NOS-2 in the CNS [60,61,62].

NO is known to induce BBB disruption. For example, blockade of NO production by Nomega-Nitro-l-arginine methyl ester, a non-specific NOS inhibitor, abolished BBB disruption following focal cerebral ischemia/perfusion in animal models [63,64]. However, the effects of NO on endothelial cell apoptosis are complicated. Shen et al. [65] showed that the anti-apoptotic effect of NO on endothelial cells was exerted through the cyclic guanosine monophosphate (cGMP) pathway, while NO induced apoptosis through cGMP-independent pathways. The effects of NO on TJ-related proteins are clearer, with a confirmed reduction in TJ-related proteins following NO production [66].

#### 3.1.4. Glutamate

Glutamate is a major excitatory transmitter and play a key role in synaptic plasticity for learning and memory, which exerts its excitatory effects via glutamatergic receptors, including the N-methyl-D-aspartate (NMDA) receptor and the α-amino-3-hydroxy-5-methyl-4-isoxazolepropionic acid (AMPA) receptor. Glutamate is not only released from neurons but also astrocytes, and astrocyte-derived glutamate acts as a gliotransmitter to nearby neurons to regulate synaptic plasticity and formation. NMDA receptors are also distributed in endothelial cells as well as neurons [67,68], and astrocyte-derived glutamate can induce vasodilatation that is dependent on NOS-3 and activation of endothelial NMDA receptors [69].

Although glutamate is essential for normal function of neurons and endothelial cells, excessive glutamate causes deleterious effects including neuronal death and BBB disruption. For example, perfusion of glutamate induced excessive vascular permeability via activation of NMDA receptors [70], while following permanent focal cerebral ischemia in rats, blockade of NMDA or AMPA receptors attenuated BBB disruption [71]. With respect to the effects of glutamate on endothelial TJ-related proteins, András et al. [68] suggested that treatment of glutamate decreased OCLN protein levels in brain endothelial cells. As excessive glutamate is released from astrocytes following brain injury, astrocyte-derived glutamate must be involved in BBB disruption via activation of endothelial glutamate receptors.

#### 3.1.5. Endothelins

Endothelins (ETs) including ET-1, -2 and -3 are potent endogenous vasoconstrictors and exert various physiological actions other than vasoconstriction including regulation of endothelial function. There are two types of ET receptors including via endothelin receptor type A (ETA ) and type B (ETB ), and ETs exert bioactive functions via ETA and ETB receptors. In patients with brain damages including TBI and subarachnoid hemorrhage, ET-1 is increased in cerebrospinal fluid and associated with unfavorable outcomes [72,73]. The production of ET-1 is performed in various types of cells in CNS. In various experimental animal models, ET-1 production was also observed in astrocytes [74,75,76], while targeted overexpression of ET-1 in astrocytes led to a higher mortality, more severe neurological deficits and cerebral edema in subarachnoid hemorrhage and transient ischemia model mice [77,78]. Hung et al. [79] also reported that selective astrocytic ET-1 overexpression exacerbated cerebral edema, neurodegeneration, neuroinflammation, oxidative stress and memory deficits in transient cerebral ischemia mice.

The involvement of ET-1 in BBB disruption is supported by experimental models in vivo and in vitro. Repeated administration of ET-1 enhanced disruption of BBB permeability in dogs and rats [80]. Reijerkerk et al. [81] also reported that ET-1 contributed to the brain endothelial barrier passage of monocytes involved in BBB inflammation via ETB receptor signaling in brain endothelial cells. ET-1 also induced upregulation of ICAM-1 and VCAM-1 expression in human brain microvascular endothelial cells [82]. Further, astrocytic overexpression of ET-1 increased the severity of BBB breakdown in subarachnoid hemorrhage mice [78]. The effects of blockade of the ET system for BBB disruption have also been examined. For example, the selective ETA receptor antagonist S-0139 reduced BBB permeability, brain edema formation and infarct size after cerebral ischemia/reperfusion in rats [83], while Kim et al. [84,85] reported that the selective ETB receptor antagonist BQ788 blocked BBB disruption via inhibition of MMP-9 activation and ZO-1 protein degradation in experimental status epilepticus animals.

### 3.2. The Vascular Protective Factors

#### 3.2.1. Angiopoietin-1

Angiopoietin-1 (ANG-1) is a glycoprotein with angiogenetic properties, which are exerted via Tie-2, a tyrosine kinase receptor expressed principally in endothelial cells. When ANG-1 binds Tie2, the cytoplasmic tyrosine residues of Tie2 is phosphorylated, resulting in activation of various intracellular signaling including Phosphoinositide 3-kinase /AKT, Ras and mitogen-activated protein kinase which are involved in the survival of endothelial cells and vascular remodeling and stability. A protective effect of ANG-1 via Tie-2 signaling in neurons after brain damage was also previously reported [86]. In CNS, endothelial cells produce ANG-1 while ANG-1 expression was also found in astrocytes in the cerebrum of experimental animals and in cultured cells [87,88,89,90,91]. A range of studies have found protective effects of ANG-1 on BBB function. Meng et al. [92] demonstrated that ANG-1 overexpression reduced BBB leakage, while exogenous ANG-1 or ANG-1 mimetic peptides suppressed BBB damage [93,94], in animal models of focal embolic cerebral ischemia. In subarachnoid hemorrhage rats, the administration of exogenous ANG-1 reduced BBB leakage [95]. In addition, blockade of Tie-2 activation exacerbated BBB disruption in TBI mice by controlled cortical impact (CCI) [96]. These observations suggest protective effects of ANG-1/Tie-2 against BBB damage. In patients with brain damages, alterations of ANG-1 level have been indicated. Plasma ANG-1 concentrations were low after ischemic stroke particularly in patients with poor stroke outcomes [97]. Sobrino et al. [98] suggested that high serum levels of ANG-1 were associated with good outcome in patients with intracerebral hemorrhage.

Interestingly, Nag et al. [99] found only minimal expression of caspase-3 after ANG-1 production by the endothelium following cortical cold injury in rats. Further, Zhao et al. [100] suggested that ANG-1 inhibited glycation end product-induced endothelial cell apoptosis. The functional effects of ANG-1 on endothelial TJ-related proteins have also been reported, with reversal of the decrease in TJ-related proteins with ANG-1 treatment following cerebral ischemia/perfusion in rats [101]. Further, Xia et al. [90] suggested that ANG-1 caused upregulation of ZO-1 and OCLN to repair TJs after permanent ischemic damage in rats. ANG-1 also suppressed VEGF-induced expression of ICAM-1 and VCAM-1, and reduced VEGF-induced leukocyte adhesion to HUVECs [41].

#### 3.2.2. Sonic Hedgehog

Sonic hedgehog (SHH) is a glycoprotein that belongs to the hedgehog family, and is essential for normal pattern formation and cellular differentiation in the developing CNS. The SHH signaling pathway is initiated by the binding of SHH to Patched-1 (PTCH1), which blocks the inhibitory action of the PTCH1 receptor to Smoothened, a membrane protein, resulting in activation of transcription factors [102]. In CNS, the production of SHH is observed in astrocytes, immune cells and endothelial cells [103]. In experimental animals and cultured cells, SHH production was predominantly observed in astrocytes [104,105,106,107,108], and astrocyte-derived SHH contributed to angiogenesis [106,107]. The beneficial effects of SHH for reducing BBB disruption have also been confirmed. Administration of recombinant SHH decreased BBB leakage in permanent ischemia model rats [90]. Furthermore, Alvarez et al. [105] showed that astrocyte-secreted SHH promoted BBB formation and integrity through endothelial hedgehog receptors.

Gao et al. [109] reported that downregulation of PTCH1 enhanced endothelial progenitor cell apoptosis induced by high glucose. Zhu et al. [110] also demonstrated that the SHH signaling pathway was protective against endothelial cells apoptosis. Therefore, SHH must exert anti-apoptotic effects via SHH signaling pathways in endothelial cells after brain damage. The effects of SHH on TJ-related proteins have also been reported. SHH or a SHH signaling agonist increased expression of CLN-5, OCLN and ZO-1 in brain endothelial cells, whereas a SHH signaling inhibitor blocked these effects [108]. Brilha et al. [54] also showed that treatment of exogenous SHH reduced the mycobacterium tuberculosis-induced BBB breakdown and reversed the decrease in CLN-5 in a co-culture BBB model consisting of brain microvascular endothelial cells and astrocytes. In permanent ischemia model rats, administration of SHH increased the expression of ZO-1 and OCLN [90]. Further, SHH reduced the levels of ICAM-1 expression in endothelial cells, and suppressed adhesion and transmigration of immune cells [105]. As a relationship of SHH for clinical disease, Drannik et al. [111] implied that SHH pathway may be compromised in ALS patients.

#### 3.2.3. Glial-Derived Neurotrophic Factor

Glial-derived neurotrophic factor (GDNF) is a neurotrophic factor secreted from astrocytes and activates GDNF receptor alpha-1 and -2 expressed in neurons and endothelial cells, resulting in survival of neurons, axon guidance and synapse formation and control of endothelial functions. It was previously reported that GDNF can promote angiogenesis [112], and that GDNF is critical for normal postnatal development of the BBB [113]. Further, Igarashi et al. and Shimizu et al. [114,115] found that GDNF treatment increased CLN-5 expression and strengthened the barrier function in brain endothelial cells. Xiao et al. [116] also confirmed upregulation of OCLN and ZO-1 by GDNF. These results imply that GDNF exerts protective effects against BBB disruption by increasing TJ-related proteins in endothelial cells.

#### 3.2.4. Retinoic Acid

Retinoic acid (RA) is an active metabolite of vitamin A, and is synthesized from retinol by retinaldehyde dehydrogenase (RALDH). RA acts as a ligand for nuclear RA receptors (RARs), which are important for growth and development in the CNS. RA are also associated with learning and memory behaviors by regulation of synaptic plasticity in the mature brain. The production of RA is observed in various types of cells including neurons and glial cells in CNS. RALDH2 is highly expressed in reactive astrocytes, which causes enhanced astrocytic RA synthesis [117].

Recent studies support a role for RA in the development and protection of the BBB. For example, Mizee et al. [118] suggested that RA is crucial for development of the brain endothelial cell barrier via RARβ signaling in the developing brain vasculature. During BBB differentiation, the inhibition of RAR activation caused leakage of serum proteins into the developing brain, and reduced the expression of BBB determinants [118]. The enhanced RA synthesis by increased expression of RALDH2 in reactive astrocytes also protected BBB function during inflammatory stimulation [117]. In addition, injection of RA increased expression of ZO-1 and vascular endothelial cadherin, which are crucial components of the BBB structure [119]. RA also reduced VCAM-1 expressions in cultured dermal microvascular endothelial cells during inflammatory conditions, and decreased VCAM-1-dependent T cell binding to microvascular endothelial cells [120]. Therefore, similar effects of RA may also exert in brain microvascular endothelial cells.

#### 3.2.5. Insulin-Like Growth Factor-1

Insulin-like growth factor-1 (IGF-1) is a member of the insulin gene family, and exerts bioactive functions as a neurotrophic factor via activation of the IGF-1receptor. IGF-1 exerts multiple physiological roles including neurogenesis, prolonged neuronal survival, reduced cell death, resistance to injury, reparation and neuroplasticity in the adult brain [121]. Downregulation of the IGF-1 receptor promoted cellular apoptosis induced by advanced glycation end products in cultured vascular endothelial cells [122]. Therefore, anti-apoptotic effects of IGF-1 against brain endothelial cells are expected.

Astrocytes are one of product cells for IGF-1 although the production of IGF-1 is also observed in neurons, endothelial cells and other glial cells [123,124], and astrocyte-derived IGF-1 plays a key role in neuronal protection after brain damage. Astrocytic overexpression of IGF-1 also protected neurons against TBI by CCI [125], while astrocyte-IGF-1 gene transfer improved outcomes in rats following ischemia/perfusion [126].

Bake et al. [127] reported that IGF-1 reduced BBB permeability and decreased infarct volume in ischemia/perfusion rats. Further, in primary brain microvessel endothelial cells exposed to stroke-like conditions by oxygen-glucose deprivation, IGF-1 reversed the excessive dye transfer across the cell monolayer [128]. These results suggest that astrocyte-derived IGF-1 exerts protective effects against endothelial cell death, thus attenuating BBB disruption.

#### 3.2.6. Apolipoprotein E

Apolipoprotein E (APOE) is a member of the apolipoprotein family which supports lipid transport and injury repair in the brain [129]. In experimental animals and humans, production of APOE is predominantly synthesized in and secreted from astrocytes in CNS [130,131,132].

Multiple studies indicate APOE is protective factor for BBB disruption in experimental animal models. In TBI mice by CCI, APOE-mimetic peptide COG1410 reduced Evans blue extravasation and suppressed the activity of MMP-9 [133]. On the other hand, the increased Evans blue extravasation was found in the brains of APOE KO mice after CCI compared with WT mice [134]. In addition, more activated MMP-9 was detected in APOE KO mice after CCI compared with WT mice while the expressions of OCLN and ZO-1 were decreased in APOE KO mice [134]. In animal models of CNS inflammation, Zheng et al. [135] suggested that APOE-deficient promoted BBB disruption, upregulated MMP-9 expression activity and decreased the expression of endothelial TJ-related proteins.

## 4. Astrocytic Molecules as Candidates for Therapeutic Strategies to Protect BBB

**Figure 3:**
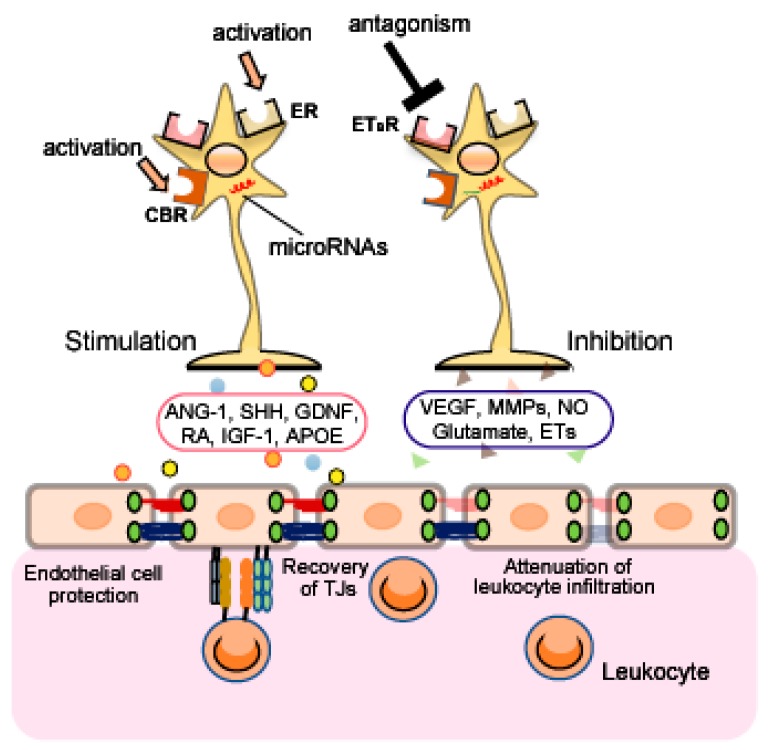
Therapeutic strategy for BBB protection and recovery of BBB function by controlling the function of astrocyte-derived factors. Astrocytic estrogen receptor (ER), ET_B_ receptor (ET_B_R), cannabinoid receptor (CBR) and microRNAs are involved in the regulation of astrocyte-derived factors production. Hence, these receptors and microRNAs are candidates for protection/recovery of BBB functions by modulating the actions of astrocyte-derived factors.

Therapeutic strategies to target astrocytes have been proposed in a range of neurodegenerative disorders [136,137,138], spinal cord injury [139], hyperalgesia [140], mental illnesses [141], TBI [142] and cerebral ischemia [143]. As astrocytes are involved in regulation of the BBB, targeting astrocytic function may protect against brain injury induced by BBB disruption. In this section, we describe several astrocytic molecules targeted for control of astrocyte function (Figure 3).

### 4.1. Estrogen Receptors

Estrogen and progesterone are known to control astrocyte functions and exert protective effects against brain damage. Arevalo et al. [144] and Acaz-Fonseca et al. [145] reported that the gonadal hormones suppressed astrogliosis and reduce neuroinflammation and brain edema after various types of CNS injury. In animal models of TBI by the Marmarou method and cerebral ischemia/perfusion, estradiol also attenuated BBB disruption [146,147,148,149]. Further, estradiol blocked the upregulation of MMPs after cerebral ischemia [150], and increased ANG-1 expression through ERα in the rat cerebrum [151]. Estradiol also inhibited induction of VCAM-1 and ICAM-1 expressions in cultured human endothelial cells during inflammatory conditions [152].

Numerous studies indicate that astrocytes express estrogen receptors (ERs) and that astrocytic ERs mediate the neuroprotective actions of estradiol [153,154,155,156]. The astrocytic ERs also regulate the production of several astrocyte-derived factors including neurotropic factors and chemokines [153,157,158]. These observations imply that activation of astrocytic ERs may be neuroprotective by alleviating BBB disruption.

### 4.2. Endothelin Receptor Type B

Although astrocytes can produce ET-1 (see Section 3.1.5.), astrocytes are also targets of ET-1. The predominant expression of ETB receptors in the brain is found in astrocytes [12,159,160]. Beneficial effects of ETB antagonist on BBB disruption have been reported in experimental animal models. For example, Kim et al. [84,85] found that the selective ETB antagonist BQ788 reduced BBB disruption in experimental status epilepticus. We also reported that BQ788 attenuated the BBB disruption and blocked the decrease in expression of TJ-related proteins after TBI in mice by FPI [12]. ETB antagonist may also ameliorate inflammatory damage because ET-1 increased in endothelial CAMs and contributed to the brain endothelial barrier passage of monocytes [81,82].

Astrocytic ETB receptors are known to control astrocyte functions. For example, activation of astrocytic ETB receptors causes astrocytes to transition from their resting form to a reactive form [12,159,160]. LeComte et al. [161] also showed that astrocyte-specific deletion of the ETB receptor causes a defect in reactive astrocyte proliferation after permanent cerebral ischemia. Further, we previously reported that activation of ETB receptors increased astrocytic MMP-9 and VEGF-A expression, and decreased astrocytic ANG-1 expression [89,162]. Therefore, blockade of astrocytic ETB receptors is an attractive candidate for repairing BBB disruption after brain injury.

### 4.3. Cannabinoid Receptors

The cannabinoid (CB) receptors, including CB1 and CB2, are a class of cell membrane G protein-coupled receptors that are activated by endocannabinoids or exogenous agonists. Numerous studies have shown a protective action of CB via CB receptors against BBB disruption and TJ-related proteins in experimental animals and cell models [163,164,165,166].

Astrocytes express CB receptors [167,168,169], and upregulation of CB receptors in reactive astrocytes was observed in animal models of amyotrophic lateral sclerosis and epilepsy [167,170]. Kozela et al. [168] also reported that increased glial fibrillary acidic protein, a marker of astrocyte activity, was suppressed by CB in various experimental animal models, suggesting that modulation of astrocytic CB receptors may have beneficial effects for treatment of brain disorders.

### 4.4. MicroRNAs

MicroRNAs (miRNAs) are small non-coding RNAs observed in the brains of humans and experimental animals, which regulate the expression of various genes under both normal and pathological conditions. The multifarious miRNAs are closely involved in both BBB disruption and protection in various experimental animal models [171,172,173,174,175]. Further, during neuroinflammation, expression of brain endothelial microRNA-125a-5p was suppressed, resulting in increased monocyte migration as a result of endothelial upregulation of ICAM-1 [176]. Recent studies suggest that astrocytes express various miRNAs, and these miRNAs control astrocytic functions [177,178,179,180,181,182]. Overexpression of miRNA-21 in astrocytes attenuated astrogliosis, while inhibition of miRNA-21 function enhanced astrocytic hypertrophy in spinal cord injury (SCI) animals [177]. Similarly, Wang et al. [183] showed that astrocyte-specific overexpression of miRNA-145 reduced astrogliosis in SCI rats. Therefore, astrocytic miRNAs are a potential therapeutic target for SCI by alleviating astrogliosis. Moreover, several studies have found that various miRNAs can regulate VEGF expression in endothelial cells in the cerebrum and in glioma cells [184,185,186]. The control of MMP expression by miRNAs was also shown following cerebral ischemia in rats, and in primary fetal astrocyte-enriched cell cultures and glioma cells [182,187,188]. As expression of these miRNAs is observed in astrocytes, a similar regulation of VEGF and MMPs may occur in astrocytes.

## 5. Conclusions

BBB disruption is commonly observed in TBI, cerebral ischemia and various CNS disorders including Alzheimer’s disease and multiple sclerosis, and results in severe secondary damage including brain edema and inflammatory changes. As current therapeutic strategies for various types of brain disorders do not sufficiently recover brain function, targeting BBB disruption is expected to be a novel therapeutic strategy for a wide range of brain disorders. The mechanisms of BBB disruption are complicated as they involve various types of cells and cell-derived factors. Numerous studies also suggest dual roles of astrocyte-derived factors for control of BBB function. Astrocyte-derived vascular permeability factors including VEGF, MMPs, NO, glutamate and ETs can increase BBB permeability, resulting in aggravation of BBB disruption. By contrast, astrocyte-derived protective factors including ANG-1, SHH, GDNF, RA, IGF-1 and APOE can attenuate the increase in BBB permeability leading to BBB protection. Because alterations of these factors are observed in TBI, cerebral ischemia and several CNS disorders in clinical practice, control of these factors may be significant. Astrocytes are a major therapeutic target for brain disorders, as numerous studies suggest that control of astrocytic functions can reduce brain injury in various experimental animal models. However, as described above, astrocyte-derived factors have both protective and detrimental actions against BBB disruption in brain disorders. Besides participation in formation of BBB, astrocyte is accepted to be a component of synapses, where astrocyte-derived factors regulate efficacy of neurotransmission. Because of these multiple functions, uncontrolled modulation of astrocytes may cause disturbance of brain functions including mentation and recognition. To avoid possible adverse actions in clinical use, selective stimulation of their beneficial actions without affecting the detrimental ones is required for the astrocyte-targeting therapy. Further investigation of mechanisms underlying astrocytic functions will lead to creation of more skillful methods for astrocytic control which can be applied to clinical use.

## Abbreviations

AMPA	α-amino-3-hydroxy-5-methyl-4-isoxazolepropionic acid
ANG-1	angiopoietin-1
BBB	blood-brain barrier
CAMs	cell adhesion molecules
CCI	controlled cortical impact
CB	cannabinoid
cGMP	cyclic guanosine monophosphate
CLN	claudin
CNS	central nervous system
ECM	extracellular matrix
ERs	estrogen receptors
ETs	endothelins
ET_A_	endothelin receptor type A
ET_B_	endothelin receptor type B
FPI	fluid percussion injury
GDNF	glial-derived neurotrophic factor
HUVECs	human umbilical vascular endothelial cells
ICAM-1	intercellular adhesion molecule-1
IGF-1	insulin-like growth factor-1
KO	knock-out
LFA-1	lymphocyte function-associated antigen 1
miRNAs	microRNAs
MMPs	matrix metalloproteinases
NMDA	N-methyl-D-aspartate
NO	nitric oxide
NOS	nitric oxide synthase
OCLN	occludin
PTCH1	Patched-1
RA	retinoic acid
RALDH	retinaldehyde dehydrogenase
RARs	retinoic acid receptors
SCI	spinal cord injury
SHH	sonic hedgehog
TBI	traumatic brain injury
TJ	tight junction
VCAM-1	vascular cell adhesion molecule-1
VEGF	vascular endothelial growth factor
VEGFR-1	vascular endothelial growth factor receptor-1
VEGFR-2	vascular endothelial growth factor receptor-2
VLA-4	very late antigen-4
ZO	zonula occluden
